# Pulmonary adverse reaction caused by furazolidone: Two case reports and a literature review

**DOI:** 10.1097/MD.0000000000039286

**Published:** 2024-08-16

**Authors:** Cong Zheng, Yao Cheng, Ping Huang, Miaomiao Zhang, Zhuochao Ren, Xiaochun Zheng, Zixue Xuan, Bin Shen, Xiuli Yang

**Affiliations:** a Department of Clinical Pharmacy, Yuyao People’s Hospital, Ningbo, Zhejiang, China; b Department of Pharmacy, Guangde Traditional Chinese Medicine Hospital, Xuancheng, Anhui, China; c Department of Pharmacy, Center for Clinical Pharmacy, Cancer Center, Zhejiang Provincial People’s Hospital (Affiliated People’s Hospital), Hangzhou Medical College, Hangzhou, Zhejiang, China; d School of Pharmacy, Hangzhou Normal University, Hangzhou, Zhejiang, China; e Department of Pulmonary and Critical Care Medicine, Geriatric Medicine Center, Zhejiang Provincial People’s Hospital (Affiliated People’s Hospital), Hangzhou Medical College, Hangzhou, Zhejiang, China; f Department of Pharmacy, The First Hospital of Jiaxing, Affiliated Hospital of Jiaxing University, Jiaxing, Zhejiang, China.

**Keywords:** adverse pulmonary reaction, case report, drug-induced lung injury, furazolidone

## Abstract

**Rationale:**

As one of the drugs used to treat *Helicobacter pylori*, furazolidone has been reported to cause gastrointestinal reactions, allergies, dizziness, and more. However, its related drug-induced lung injury has been rarely reported. Furthermore, there have been no reports of the timing for initiating hormone therapy when a pulmonary adverse reaction occurs.

**Patient concerns:**

We report 2 cases, both of them showed interlobular septal thickening and nodules on the chest computed tomography. One had more discomfort symptoms and had a higher eosinophil count than the normal range, while the other only had fever symptoms and had an eosinophil count within the normal range.

**Diagnoses:**

Pulmonary adverse reaction caused by furazolidone was diagnosed.

**Interventions:**

Furazolidone was discontinued, and the person with increased eosinophils received hormone therapy, while the other person did not.

**Outcomes:**

After discontinuation of medication and treatment, the symptoms of the 2 patients gradually improved.

**Lessons:**

This report suggests that furazolidone may cause pulmonary adverse reactions to raise clinical awareness, and for the first time indicates that hormone therapy is needed for patients whose eosinophils continue to increase after discontinuation.

## 1. Introduction

Furazolidone is currently mainly used for *Helicobacter pylori* (HP) infection. Currently, the quadruple regimen of PPI, bismuth and 2 antibiotics (tetracycline, metronidazole, amoxicillin, clarithromycin, levofloxacin) is the most commonly used first-line treatment for HP in China.^[1]^ A meta-analysis of 157 studies on resistance to Helicobacter pylori in Chinese adults showed that resistance rates to metronidazole, clarithromycin, and levofloxacin in Eastern China were 83.7%, 20.2%, and 20.6%, respectively, with resistance rates higher than 15% being a common threshold for choosing alternative empirical regimens.^[2]^ Although tetracycline has a low drug resistance rate of about 1.9% in Eastern China,^[2]^ clinical accessibility is poor, and because tetracycline can cause tooth staining and bone marrow suppression, it is rarely used in clinical practice.^[3]^ In the past 10 years, the resistance rate of HP to furazolidone has remained at a relatively low level.^[4]^ Furazolidone is recommended as a treatment option in the quadruple eradication regimen for Helicobacter pylori in China.^[5]^ Therefore, considering the local drug resistance, availability of clinical drugs and drug economy, furazolidone is still one of the best options for local treatment of HP. The drug instructions and previous reports of furazolidone showed that oral furazolidone tablets can cause including nausea, vomiting, diarrhea, and other gastrointestinal reactions, as well as asthma, orthostatic hypotension, hypoglycemia, lung infiltration, and occasionally hemolytic anemia and multiple neurites. However, the related pulmonary toxicities have rarely been reported. Here, we report 2 patients who developed adverse pulmonary reactions during the treatment of HP infection with a quadruple regimen containing furazolidone tablets. This study emphasizes the need for close monitoring of patients during furazolidone administration to improve drug safety.

## 2. Case reports

### 2.1. Case no. 1

**Figure 1.**
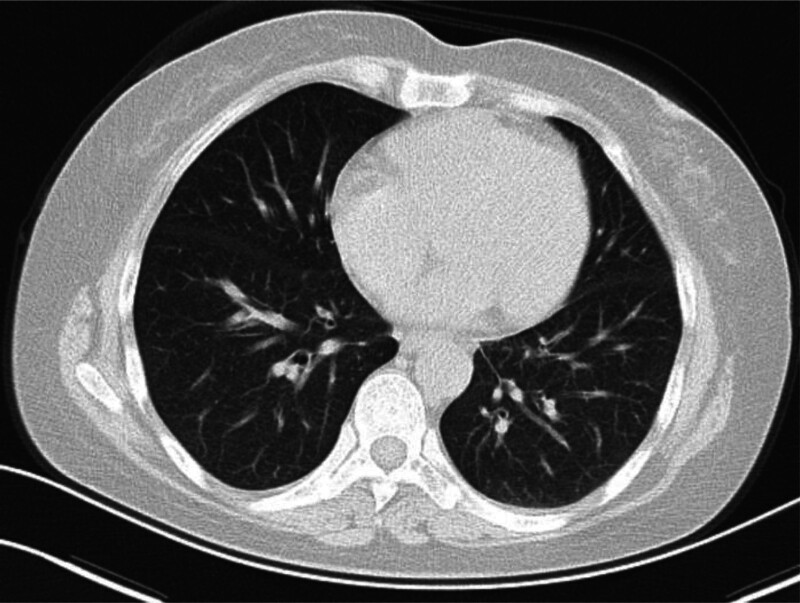
Chest computed tomography scan shows a few inflammatory and fibrous foci in both lungs and pulmonary interstitial inflammatory changes with thickening of interlobular septa.

A 51-year-old woman with no genetic history tested positive for HP during a physical examination on February 10, 2022. Chest computed tomography (CT) revealed slightly increased bilateral lung markings, clear structures of both pulmonary hili, and normal density. From February 16, 2022, oral administration of rabeprazole sodium enteric-coated capsule + colloidal bismuth pectin capsules + amoxicillin capsules + furazolidone tablets (100 mg, twice daily) for the treatment of HP infection was initiated. On February 25, 2022, the patient developed a fever, with the highest body temperature reaching 38.9°C, cough, and expectoration accompanied by chest tightness and shortness of breath; consequently, the quadruple anti-HP treatment was discontinued. A routine blood examination at a local hospital showed a white blood cell (WBC: 3.5–9.5 × 10^9^/L) count of 8.7 × 10^9^/L, neutrophil percentage (N%: 40–75%) of 89.9%, C-reactive protein (CRP: 0.0–10.0 mg/L) level of 9.1 mg/L, and eosinophil (EO: 0.02–0.52 × 10^9^/L) count of 0.05 × 10^9^/L. After treatment with a cold medicine and anti-inflammatory drugs (specific medication details are unclear), the patient’s fever and other symptoms improved but recurred. On March 2, 2022, because of fever, cough, sputum, chest tightness, shortness of breath, and other nighttime symptoms, she visited our hospital for treatment. Chest CT examination showed a few inflammatory and fibrous foci in both lungs and pulmonary interstitial inflammatory changes with thickening of the interlobular septa (Fig. 1). Routine blood examination showed an increase in WBC count at 17.98 × 10^9^/L, N% at 88.6%, CRP level at 87.8 mg/L, and EO count at 0.61 × 10^9^/L. Auscultation of the bilateral lung sounds was harsh, although no rales were heard. On March 3, 2022, the patient was admitted to the Department of Respiratory Medicine and administered the following treatment regimen: intravenous 2 g cefoperazone sodium sulbactam sodium injection every 8 hours for antibiotic activity; atomization inhalation of a suspension of 5 mg terbutaline, 2 mg budesonide, and 0.3 g acetylcysteine inhalation solution twice daily; and 30 mg ambroxol hydrochloride injection via a micropump twice daily. The patient experienced relief after this treatment. On March 4, 2022, a routine blood examination showed the following: WBC count 6.01 × 10^9^/L, N% 45.7%, CRP level 56.4 mg/L, and EO count 0.63 × 10^9^/L. On March 7, 2022, blood routine examination showed a WBC count of 5.77 × 10^9^/L, N% 55.6%, CRP level 6.0 mg/L, and EO count 0.33 × 10^9^/L. Combined with the patient’s medication history, laboratory test and lung imaging, the clinician ruled out bacteria, viruses, fungi, *Pneumocystis carinii* pneumonia, connective tissue diseases, and other possibilities, believed that the patient to be drug-induced lung injury.

### 2.2. Case no. 2

**Figure 2.**
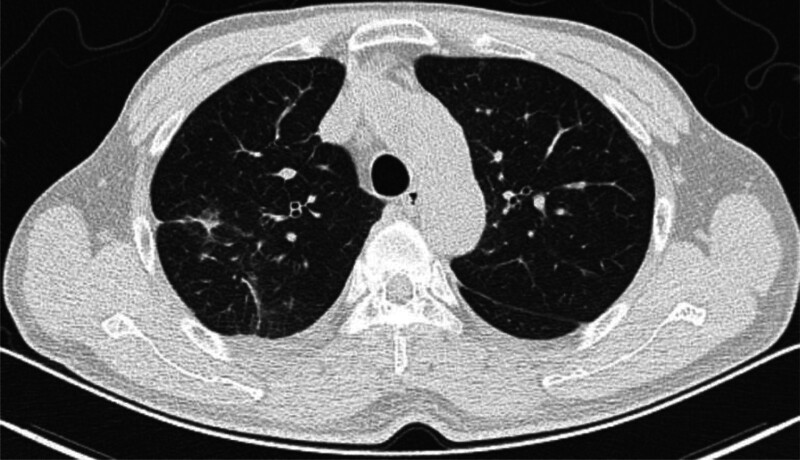
Chest computed tomography scan shows a small amount of inflammation, some fibrous foci in both lungs, and interstitial changes in both lungs with interlobular septal thickening.

A 48-year-old man with a 15-year history of diabetes mellitus was under regular hypoglycemic therapy. On March 28, 2022, the patient underwent gastroscopy, and the pathological results showed HP positivity. From April 6, 2022, rabeprazole sodium enteric-coated capsules + bismuth potassium citrate capsules + amoxicillin capsules + furazolidone tablets (100 mg, twice daily) were administered orally to treat HP infection. On April 17, 2022, the patient developed symptoms of headache, general muscle ache, fatigue, chills, and a body temperature of 38°C, for which he visited our hospital. A routine blood examination showed an increased WBC count of 13.16 × 10^9^/L, N% of 83.2%, CRP level of 70.2 mg/L, EO were normal, with a count of 0.13 × 10^9^/L. Amoxicillin and furazolidone tablets were discontinued based on clinical advice and a daily 100 mL intravenous levofloxacin injection via drip was initiated for anti-infection treatment. On April 18, 2022, the patient’s body temperature returned to normal. He revisited the emergency department of our hospital to continue the intravenous infusion of levofloxacin. While amoxicillin was still discontinued in accordance with medical advice, he resumed taking furazolidone, after which he developed a fever again, with a high temperature of 39.2°C. The patient visited our hospital again on April 19 and was prescribed a one-time 1 g intravenous ertapenem injection via drip for anti-infection treatment. Additionally, chest CT showed a small amount of inflammation, some fibrous foci in both lungs, and interstitial changes in both lungs with interlobular septal thickening (Fig. 2). Prior to this, the patient’s most recent chest CT scan was on August 13, 2018, which revealed scattered chronic inflammatory lesions (mainly fibrous lesions) in the right lung. The physician consulted a clinical pharmacist, who, considering that the patient’s symptoms improved after stopping furazolidone on April 17 and worsened after reapplication on April 18, which had a significant time correlation, reminded the physician to consider the possibility of adverse pulmonary reactions caused by furazolidone, and recommended the patient to discontinue furazolidone tablets. On April 20, 2022, the patient’s body temperature was 37.4°C. The breath sounds were thick upon auscultation of both lungs, with no wet or dry rales or wheezing. A routine blood examination showed an increased WBC count of 9.86 × 10^9^/L, N% of 76.8%, CRP level of 223.7 mg/L, EO were still normal, with a count of 0.35 × 10^9^/L; hence, he was admitted to the Department of Respiratory Medicine and his temperature was measured at 36.8°C. After admission, an intravenous infusion of 0.5 g meropenem was administered every 6 hours, starting at approximately 21:00 hours. Improved laboratory test results accompanied by lung imaging led to the exclusion of bacteria, pulmonary viruses, tuberculosis, and fungi.

On April 21, 2022, procalcitonin level increased to 0.67 ng/mL. Considering the patient’s history of diabetes mellitus, the possibility of infection in other parts of the body could not be ruled out. Moreover, given that the patient’s symptoms, such as fever and other signs of discomfort, were relieved after discontinuing furazolidone administration, the patient continued to receive only anti-infection treatment. The WBC count, CRP level, and N% decreased continuously during hospitalization. On April 25, 2022, the blood results were as follows: WBC count: 6.28 × 10^9^/L, N%: 50.3%, CRP level: 13.5 mg/L, EO count: 0.44 × 10^9^/L, and procalcitonin: 0.11 ng/mL. The patient was discharged after his symptoms improved.

## 3. Discussion

### 3.1. Evaluation of relevance

Case no. 1 was considered to be drug-induced pulmonary adverse reactions after excluding other disease. However, after literature review, there have been no reports of pulmonary adverse reactions caused by rabeprazole, colloidal bismuth pectin capsules and amoxicillin, while there have been limited reports on furazolidone, therefore, the occurrence of pulmonary adverse reactions may not be related to rabeprazole, colloidal bismuth pectin capsules and amoxicillin. After reviewing the relevant literature on pulmonary adverse reactions caused by furazolidone, we found that the main manifestations of pulmonary toxicity caused by furazolidone included fever, cough and sputum, pulmonary interstitial infiltration, and elevated eosinophilic counts, which were consistent with the adverse reactions in this patient, so we considered the possibility of pulmonary adverse reactions caused by furazolidone.

The Nord’s Adverse Drug Reactions (ADR) Assessment Scale^[6]^ consists of ten medical questions related to ADR with predetermined scores (Table 1), mainly used to evaluate and determine the correlation and degree of closeness between drug use and ADR. According to the Assessment Scale, the total scores for Case no. 1 were 7 points (Table 2, a total score of 5–8 points is likely to be related).

**Table 1:** Nord’s ADR Assessment Scale.

Related issues	Question score		
	Yes	No	Unknown
1. Whether the ADR has a previous conclusive report?	+1	0	0
2. Whether the ADR occurred after the use of the suspect drug?	+2	−1	0
3. Whether the ADR resolves after discontinuation or application of an antagonist?	+1	0	0
4. Whether the ADR recurred after repeated use of the suspect drug?	+2	−1	0
5. Whether there are other reasons that can cause the ADR alone?	−1	+2	0
6. Whether the ADR recurred after placebo was administered?	−1	+1	0
7. Whether the drug has reached toxic concentrations in the blood or other body fluids?	+1	0	0
8. Whether the ADR worsens with increasing dose or resolves with decreasing dose?	+1	0	0
9. Whether the patient has been exposed to the same or similar drug and had a similar reaction?	+1	0	0
10. Whether there is any objective evidence to substantiate the reaction?	+1	0	0

Note: If the total score is ≥9 points, it indicates that the causal relationship between the drug and adverse reactions is positive, which is confirmed by objective evidence and quantitative testing data; A total score of 5 to 8 points is likely to be related, that is, supported by objective evidence or quantitative testing results; A total score of 1 to 4 points may be related, indicating that it cannot be fully confirmed and cannot be a completely negative situation; If the total score is ≤0, it is considered suspicious, which belongs to accidental or basically unrelated situations.

**Table 2:** Nord’s ADR Assessment Scale for furazolidone caused pulmonary adverse reactions in 2 patients.

Related issues	Scoring situation					
	Case no. 1			Case no. 2		
	Yes	No	Unknown	Yes	No	Unknown
Question 1	+1			+1		
Question 2	+2			+2		
Question 3	+1			+1		
Question 4			0	+2		
Question 5		+2			+2	
Question 6			0			0
Question 7			0			0
Question 8			0			0
Question 9			0			0
Question 10	+1			+1		
Total score	7 points			9 points		

Case no. 2 developed fever and other uncomfortable symptoms on the 12th day after the application of furazolidone, and the temperature returned to normal after stopping the use of furazolidone, and fever again occurred on the 13th day after the use of furazolidone as instructed by the doctor. Subsequently, after stopping the use of furazolidone on the 14th day, the symptoms including fever gradually improved until recovery. According to the Nord’s ADR Assessment Scale, the total scores for Case no. 2 were 9 points (Table 2, the total score of ≥9 points is definitely to be related).

### 3.2. Characteristics of furazolidone-induced adverse pulmonary reactions

Drug-induced lung injury is defined as lung injury caused by the use of specific drugs. It is an adverse reaction that occurs specifically in the lungs, bronchi, pulmonary vessels, and pleura. The main manifestations include inflammation and fibrosis of the alveoli, pulmonary interstitium, and small pulmonary vessels.^[7]^ However, a literature search yielded limited information on furazolidone-induced adverse pulmonary reactions, with only 4 published articles (Table 3).^[8–11]^.

**Table 3:** Characteristics of the cases of furazolidone-induced adverse pulmonary reactions from the literature.

Sex, age (yr)	Dosage of furazolidone	Time of occurrence	The main symptoms	Eosinophil status	Other auxiliary examinations	Radiological manifestations	Treatment measures	Recovery time
Male,^[8]^ 50	100 mg, twice daily	Adverse reactions occurred on day 4 after taking the drug	Fever (max: 39.4°C), dyspnea, chest tightness, headache and cold tremor	Progressive elevation in the eosinophil count after drug discontinuation and increased to 11% after 7 days of discontinuation (after hormone treatment)	WBC: 10.3 × 10^9^/L↑	Chest radiograph showed diffuse bilateral acute infiltration	Inhalation therapy, bronchodilators, expectorants and veins doxycycline. When the disease worsened, hydrocortisone 200 mg IVGTT and prednisone 15 mg oral treatment were administered for 3 days	On the third day of treatment (hormone induced use the next day)
Male,^[9]^ 56	Not mentioned	Medicine was administered for 5 days, and adverse reactions occurred 2 days after discontinuation	Fever (maximum body temperature unknown), dyspnea	Progressive increase in the eosinophil count after drug discontinuation and increased to 1.05 × 10^9^/L↑ after 69 days of discontinuation (before hormone treatment)	WBC: 17.5 × 10^9^/L↑, lymphocyte transformation test tested positive to furazole strong ketone positive	Chest radiograph showed double diffuse mottling of the lung	Flucloxacillin was used for 11 days, and the patient was hospitalized after symptom aggravation	Seven days after admission, chest radiograph showed the disappearance of diffuse mottling of both lungs
Female,^[10]^ 43	125 mg, 4 times daily	Medication was taken on days 1–10 and 13, and adverse reactions occurred on day 14	Fever (max: 38.6°C), dyspnea, chest pain, and headache	Progressive increase in the eosinophil count after drug discontinuation and increased to 21% after 3 days of discontinuation (before hormone treatment)	WBC: 15.8 × 10^9^/L↑	Chest radiograph showed bilateral lower lobe and left lung interstitial infiltrates	Oxygen treatment, levofloxacin 500 mg, and doxycycline 100 mg twice daily; prednisone 40 mg once daily was administered when the disease became worse, and the course of treatment was more than 1 mo	Dyspnea improved rapidly and abated on day 3 of treatment (day of hormone administration)
Female,^[11]^ 38	100 mg, twice daily	Adverse reactions occurred on day 11 after taking the drug	Absence of fever, progressive fatigue, cough	Eosinophil count gradually increased to 0.55 × 10^9^/L (before hormone treatment)	ESR: 44 mm/h↑, IgE: 966 IU/mL↑	Chest CT revealed bilateral interstitial infiltrates, mainly manifested as interlobular septal thickening and nodules	Intravenous prednisone (40 mg/d) for 6 days, and then switched to oral hormone therapy, and the dose was gradually reduced over a week	The symptoms rapidly subsided after treatment (the specific recovery time was not mentioned)
Female,^[11]^ 36	100 mg, twice daily	Adverse reactions occurred on day 11 after taking the drug	Fever (max: 38.5°C), cough	Eosinophil ratio gradually increased to 9.8% (before hormone treatment)	CRP: 11.2 mg/L↑	Chest CT showed bilateral interstitial infiltrates, including patchy hyperdense foci, combined with thickening of the interlobular septa and nodules	Antipyretic treatment (specific drug unknown)	The symptoms improved rapidly (the specific recovery time was not mentioned)

↑ = increase, CRP = C-reactive protein, CT = computed tomography, ESR = erythrocyte sedimentation rate, IgE = immunoglobulin E, WBC = white blood cells.

Based on the literature and 2 cases of adverse pulmonary reactions at our hospital, furazolidone initiated symptoms of discomfort 4 to 14 days after administration. All 7 cases were discontinued furazolidone eventually, and 6 of them underwent hospitalization. Lung imaging revealed bilateral interstitial infiltrates. However, since only the radiographs of the 3 patients have been shown in the past, the specific imaging findings of the chest CT are unknown. Four patients including our 2 patients showed interlobular septal thickening and nodules on the chest CT. Six patients had eosinophils higher than normal, among these 6 patients, besides fever symptoms, there are also other different symptoms, such as 4 had dyspnea and headache; 3 had cough; 2 had chest pain; 2 had fatigue; and one each had chills, muscle soreness, and rash. However, another patient with normal eosinophils only had fever symptoms. Among the 7 patients, 5 patients were administered empirical antibiotics because of the high fever and inflammatory indices. The conditions of 4 of these patients with eosinophil persistent elevation worsened after antibiotic treatment and experienced rapid, significant, sustained relief in symptoms after receiving steroid treatment. Another study did not report specific treatment drugs, therefore, it is unknown whether a follow-up steroid treatment was administered. Among the rest 2 patients who did not take hormone therapy, one patient’s eosinophils gradually decreased and returned to normal after discontinuation of the drug. Another outpatient stopped taking furazolidone on the first day of symptoms, and the eosinophils were not reviewed, so it is unknown the trend of her eosinophils after stopping the medication. Additional details are present in Table 3.

### 3.3. A possible mechanism underlying furazolidone-induced adverse pulmonary reactions

The mechanism underlying furazolidone-induced adverse pulmonary reactions remains unclear. Furazolidone is reported to cause pulmonary hypersensitivity. Furazolidone and nitrofurantoin are considered to have similar chemical structures, and the diffuse lung toxicity caused by nitrofurantoin is one of the most common lung toxicities reported thus far.^[12]^ Common manifestations of nitrofurantoin-induced pulmonary toxicity are lung infiltration, dyspnea, shortness of breath, and chest pain. Other features include sudden onset and rapid clearance, late eosinophilia, and the efficacy of glucocorticoid treatment in alleviating symptoms. Thus, nitrofurantoin and furazolidone are highly similar in terms of adverse pulmonary reactions. Nitrofurantoin contains a 5-nitrofuran ring that can act as a prodrug and must be activated by nitro reduction. However, nitro reduction can cause toxicity, including DNA damage, oxidative stress, and inhibition of RNA and protein biosynthesis. Symptoms, such as excitement, tremors, convulsions, peripheral neuritis, gastrointestinal irregularities, poor weight gain, depression, and alcohol intolerance, may also occur. Because of the similar energy required for reduction, all nitrofurantoins can be reduced to type I or type II nitrofurantoins, wherein reactive and carcinogenic hydroxylamine is produced; the reactive oxygen species produced by the redox cycle during type II nitro reduction has pharmacological effects. One study reported reduced lung tissue damage when nitrofurantoin and antioxidants were used together, compared to when nitrofurantoin was used alone.^[13]^ The study suggests that oxidation is responsible for pulmonary toxicity. Similar to nitrofurantoins, furazolidone contains a 5-nitrofuran ring. Thus, it can be reasonably speculated that furazolidone may have a type II nitro reduction effect, resulting in pulmonary toxicity. However, the specific mechanisms underlying furazolidone-induced adverse pulmonary reactions require further investigation.

### 3.4. Clinical recommendations

A furazolidone-containing regimen is commonly used to eradicate HP in patients with clinical infections, and the treatment course is usually 14 days. According to the literature and the 2 patients reported in this study, adverse pulmonary reactions may occur within 14 days of treatment, which may lead to hospitalization. Owing to the lack of characteristic symptoms and confusion between lung imaging changes and pulmonary infection, it is easy to ignore the possibility of adverse pulmonary reactions caused by furazolidone in clinical practice, resulting in antibiotic overuse. Therefore, it is necessary to timely identify the pulmonary adverse reactions caused by furazolidone.

The 5 previously reported cases only described the characteristics of pulmonary adverse reactions caused by furazolidone. Our study also found the impact of eosinophil progression on treatment outcomes from the analysis of the 7 case characteristics. We found that patients with elevated eosinophils had more severe respiratory symptoms than those with normal eosinophils. Moreover, if eosinophils gradually increase after discontinuation of medication, respiratory symptoms will continue or even worsen and hormone therapy is needed. Patients with normal eosinophils or those with eosinophils returning to normal after discontinuation of medication will quickly disappear from respiratory symptoms. In addition, we found that the time of discontinuation of medication may also be an important factor affecting treatment outcomes. Patients who discontinue furazolidone at the first sign of symptoms will experience rapid improvement in their symptoms. If used repeatedly, it can lead to rapid deterioration of the disease.

Therefore, we recommend that clinical pharmacists conduct drug reorganization in patients when faced with a similar situation as described in this study in clinical practice; moreover, diligently recording and registering patients’ past medication history is equally important. Clinicians should also be alert to such adverse reactions and actively identify the differences in lung imaging. In clinical practice, timely intervention should be administered to these patients and timely drug withdrawal should be performed. Patients who exhibited persistent elevation of eosinophil counts beyond normal levels following cessation of furazolidone treatment should be treated with hormones to prevent deterioration of their condition and promote rational drug use.

## 4. Conclusion

This study emphasizes the need for close monitoring of possible pulmonary adverse reaction when using furazolidone. This ADR can be considered based on medical history, related symptoms, eosinophilia, and changes in chest imaging. In clinical treatment, it is necessary to timely identification and cessation of furazolidone, and for patients with continuously elevated eosinophil counts, it is worth considering starting hormone therapy to avoid drug-induced damage. In addition, the conclusions proposed in this article are only based on the summary of 7 patients, and further verification is needed in the future.

## Acknowledgments

We would like to thank Dr Leixiang Yang at Zhejiang Provincial People’s Hospital (Affiliated People’s Hospital), Hangzhou Medical College for her English corrections.

## Author contributions

**Conceptualization:** Bin Shen, Xiuli Yang.

**Data curation:** Ping Huang.

**Investigation:** Miaomiao Zhang, Zhuochao Ren.

**Supervision:** Xiaochun Zheng.

**Writing – original draft:** Cong Zheng, Yao Cheng.

**Writing – review & editing:** Zixue Xuan.

## References

[1] Helicobacter pylori Study Group, Chinese Society of Gastroentterology, Chinese Medical Association. 2022 Chinese national clinical practice guideline on Helicobacter pylori eradication treatment. Chin J Dig. 2022;42:745–56.

[2] Zhou Y Hao Q Bai F-H. Meta-analysis of antimicrobial resistance of Helicobacter pylori isolates in Chinese adults. Inter J Epidemiol Infect Dis. 2024;51:49–55.

[3] Bai P Zhou LY Xiao XM Luo Y Ding Y. Susceptibility of helicobacter pylori to antibiotics in Chinese patients. J Dig Dis. 2015;16:464–70. 26147515 10.1111/1751-2980.12271

[4] Li M Zheng S. Epidemiology of helicobacter pylori resistance. Gastroenterology. 2019;24:47–50.

[5] Chinese Medical Association, Journal of the Chinese Medical Association, General Practice Branch of the Chinese Medical Association, . Guidelines for the diagnosis and treatment of Helicobacter pylori infection at the grassroots level (2019). Chin J Gen Pract. 2020;19:397–402.

[6] Chen J Qian P Cao K. Comparison and analysis of the correlation evaluation methods for adverse drug reactions in China and the Knoop assessment scale method. Chin Pharm Affairs. 2020;34:988–92.

[7] Kuboa K Azumab A Kanazawa M. Consensus statement for the diagnosis and treatment of drug-induced lung injuries. Respir Investig. 2013;51:260–77. 10.1016/j.resinv.2013.09.001 24238235

[8] Cortez LM Pankey GA. Acute pulmonary hypersensitivity to furazolidone. Am Rev Respir Dis. 1972;105:823–6. 5020630 10.1164/arrd.1972.105.5.823

[9] Collins JV Thomas AL. Pulmonary reaction to furoxone. Postgrad Med J. 1973;49:518–20. 4793499 10.1136/pgmj.49.573.518 PMC2496157

[10] Kowalski TJ Henry MJ Zlabek JA. Furazolidone-induced pulmonary hypersensitivity. Ann Pharmacother. 2005;39:377–9. 15644484 10.1345/aph.1E080

[11] Ye Y Shi Z-L Ren Z-C Sun Y-L. Furazolidone-induced pulmonary toxicity in Helicobacter pylori infection: two case reports. World J Clin Cases. 2023;11:2832–8. 37214582 10.12998/wjcc.v11.i12.2832 PMC10198096

[12] Almeida P Seixas E Pinheiro B Ferreira P Araújo A. Consider nitrofurantoin as a cause of lung injury. Eur J Case Rep Intern Med. 2019;6:1. 10.12890/2019_001295 PMC6886637 31890712

[13] Speirs TP Tuffin N Mundy-Baird F. Long-term nitrofurantoin: an analysis of complication awareness, monitoring, and pulmonary injury cases. BJGP Open. 2021;5:BJGPO.2021.0083. 34407964 10.3399/BJGPO.2021.0083 PMC9447296

